# Comparative evaluation of histopathological lesions and viral antigen distribution in domestic pigs and wild boar inoculated intranasally with the highly virulent ASFV genotype II strain “Armenia 2007”

**DOI:** 10.1186/s13567-025-01701-x

**Published:** 2026-01-06

**Authors:** Emil Wikström-Lassa, Dolores Gavier-Widén, Fabian Z. X. Lean, Alejandro Núñez, Bjørnar Ytrehus, Karl Ståhl, Pedro J. Sánchez-Cordón, Aleksija Neimanis

**Affiliations:** 1 Department of Pathology and Wildlife Diseases, Swedish Veterinary Agency (SVA), 751 89 Uppsala, Sweden; 2 Department of Animal Biosciences, Swedish University of Agricultural Sciences (SLU), Box 7028, 750 07 Uppsala, Sweden; 3 Pathology and Animal Sciences Department, Animal and Plant Health Agency (APHA-Weybridge), Woodham Lane, New Haw, Addlestone, KT15 3NB UK; 4 Norwegian Veterinary Institute, Ås, Norway; 5 Department of Disease Control and Epidemiology, Swedish Veterinary Agency (SVA), 751 89 Uppsala, Sweden; 6 Present Address: Department of Infectious Diseases and Public Health, Jockey Club College of Veterinary Medicine and Life Sciences, City University of Hong Kong, 506, Block 1A, 5/F, To Yuen Building, To Yuen Street, Kowloon, Hong Kong SAR; 7 Present Address: Department of Infectious Diseases and Global Health, Centro de Investigación en Sanidad Animal (CISA), Instituto Nacional de Investigación y Tecnología Agraria y Alimentaria (INIA), Consejo Superior de Investigaciones Científicas (CSIC), Valdeolmos, Madrid, Spain

**Keywords:** African swine fever virus, domestic pigs, wild boar, *Sus scrofa*, histopathology, virus antigen distribution, immunohistochemistry

## Abstract

African swine fever (ASF) is a devastating viral disease that affects domestic pigs (*Sus scrofa domesticus*) and European wild boar (*Sus scrofa scrofa*). A previous study indicated that wild boar were more susceptible to ASF than pigs, with shorter incubation periods and earlier onset of the disease. This follow-up study aims to compare the pathogenesis and progression of the disease between the two subspecies during the early and late stages of infection following intranasal inoculation with the highly virulent genotype II strain ‘Armenia 2007’. Histopathological changes and viral antigen distribution by immunohistochemistry (IHC) were assessed over time following intranasal inoculation. Viral antigen and histological changes were detected earlier in wild boar than in pigs. In wild boar, the medial retropharyngeal (MRPLN) and submandibular lymph nodes were among the earliest sites of virus replication from 3 days post-infection (dpi), with widespread dissemination occurring by 5 dpi. In pigs, the viral antigen was first detected in the MRPLN at 5 dpi. At the humane endpoint, which occurred at 6 dpi in wild boar and 9 dpi in pigs, virus antigen and histopathological scores were lower in wild boar than in pigs, even though the appearance and increase of viral antigen in tissues, onset and development of lesions and humane endpoint occurred earlier in the former. The lower severity and lesser extent of lesions in wild boar suggest lower tolerance of tissue damage prior to reaching the humane endpoint, demonstrating their greater susceptibility to and lower resistance against ASFV.

**Supplementary Information:**

The online version contains supplementary material available at 10.1186/s13567-025-01701-x.

## Introduction

African swine fever (ASF), caused by African swine fever virus (ASFV), which is the sole member of the Asfarviridae family [1, 2], is typically a fatal haemorrhagic disease that affects pigs (all *Sus scrofa*) and many other *Sus* species [3, 4]. ASF is currently one of the biggest threats to the global pig industry due to its high mortality rate and the high resistance of the virus, which allows it to spread easily [5–7]. The absence of treatments or globally licensed commercial vaccines [8] also makes ASF a significant animal welfare problem in both domestic pigs (*Sus scrofa domesticus*), hereafter referred to as pigs, and wild boar (*Sus scrofa scrofa*.).

The ASFV strains currently circulating in European, Asian and Caribbean countries are derived from the highly virulent genotype II strains that entered Georgia in 2007 from south-eastern Africa [9]. Despite the important role of wild boar as reservoirs and mediating the spread of the virus in Europe and Asia [10, 11], most of the data on the pathology, pathogenesis and host-virus interactions related to ASFV infection are derived from pigs [12–18] and, to a lesser extent, from wild boar [4, 15, 19–24]. As such, the knowledge of ASF for wild boar generally is inferred from the pig. Experimental data comparing these two subspecies are limited, and frequently are constrained by differences in experimental design, including age of the animals, doses, route of inoculation, virulence of the strains used or clinicopathological parameters evaluated. In vivo studies have shown that wild boar might be more susceptible to infection with virulent genotype II strains than pigs following oronasal infection [15, 18, 23]. However, these studies did not carry out sequential, predetermined culling of both pigs and wild boar at different times after infection, as required for accurate and rigorous comparative studies of pathology and pathogenesis. To date, only two studies, including our previous study, have used this experimental approach with highly virulent [25] or moderately virulent isolates [26], which also indicated that wild boar were more susceptible to ASFV than pigs.

Direct translation of the pathobiology of ASF in pigs to wild boar may therefore be problematic and represents a clear research gap. Elucidating the differences between pigs and wild boar regarding pathogenesis and mechanisms of immune response to the ASFV is essential to better understand the epidemiology and dynamics of the disease. This knowledge subsequently is needed for the development of reliable challenge models for vaccines or therapeutic assessments in these subspecies. Routine histopathological evaluations, in combination with immunohistochemical techniques used for the detection of viral antigens, can provide critical information for understanding disease dynamics and host-virus interactions.

Our previous study on clinical, gross pathological and virological findings following experimental intranasal inoculation with a highly virulent genotype II strain (Armenia 2007) suggested that wild boar had a more rapid disease progression. This included a shorter incubation period, earlier viremia and onset of clinical signs, and more rapid development of macroscopic haemorrhagic lesions than pigs [25]. The aim of this study is to further describe these differences in order to improve the understanding of disease mechanisms at the tissue and cellular level. To do this, we characterise, quantify and compare the histopathological changes and virus antigen distribution in pigs and wild boar using a standardised microscopic scoring protocol to describe disease progression from early stages of infection to humane endpoint.

## Materials and methods

### Experimental design

Details of the experimental design (animals, virus strain, dose and route of inoculation), the sequential predetermined culling of pigs and wild boar, the clinical and macroscopic evaluations performed as well as the sampling and quantification of viral DNA by qPCR in blood, swabs (rectal and nasal) and tissues have been described previously [25]. Briefly, thirty-eight animals (19 pigs aged 10–12 weeks and 19 wild boar aged 16–18 weeks) were randomly allocated into four groups consisting of 8 animals each (either wild boar or pigs), which were assigned as the infected groups. Two further groups, consisting of either 3 pigs or 3 wild boar, were assigned as non-infected controls. Before inoculation, the animals were assigned to predetermined time points at which they would be euthanised following infection. The animals were sedated and then intranasally inoculated with the ASFV virulent isolate “Armenia 2007” (genotype II). On days 1, 2, 3, and 5 post-infection (dpi), six animals (3 pigs and 3 wild boar) were sedated and euthanised each day. The remaining inoculated animals (4 pigs and 4 wild boar) were euthanised and examined by necropsy upon reaching the predetermined humane endpoint. This corresponded to 6 dpi for all remaining wild boar and 9 dpi for all remaining pigs. Pig DP38 did not reach the humane endpoint but was euthanised with its stablemates to prevent single housing. The control animals (*n* = 6) were euthanised at the conclusion of the experiment (12 dpi). Necropsy was performed on all animals, and macroscopic lesions were assessed following a standardised scoring system [27].

### Histopathological and immunohistochemical evaluations

During necropsies, an extensive suite of tissue samples from each animal was collected, fixed for 7 days in 10% buffered formalin solution, routinely processed, and embedded in paraffin blocks. To follow disease progression, organs from different systems and their regional lymph nodes (LNs) were grouped according to their location as follows: (a) Oronasal tract (nasal mucosa, palatine and pharyngeal tonsils, retropharyngeal and submandibular LN); (b) Lower respiratory tract (trachea, right cranial and caudal lung lobes, tracheobronchial LN); (c) Hepatobiliary tract (liver, gallbladder, gastrohepatic LN); (d) Intestinal tract (distal ileum, ileocaecal valve, colon, ileocaecal LN); (e) Urinary tract (kidney, urinary bladder, renal LN); (f) Other lymphoid organs (spleen, thymus, bone marrow) and g) Integument (skin). Serial tissue sections obtained from paraffin blocks were stained with haematoxylin and eosin (HE) and also used for immunohistochemical detection of ASFV antigen using a monoclonal antibody against p30/CP204L virus protein (kindly provided by Dr Linda Dixon, The Pirbright Institute, Pirbright, UK) following previously described protocols [27]. The sections were evaluated microscopically by a veterinary pathologist who was blinded to sample identity and group assignment, using previously documented histopathological and immunohistochemical scoring systems [27]. In brief, histopathological changes and the presence of cells immunolabelled for viral antigen were evaluated in different components of each organ, based on pathological evaluation criteria combined with a semi-quantitative scoring system: (0) no histopathologic changes/no presence of immunolabelled cells; (1) minimal histopathologic changes/occasional presence of immunolabelled cells; (2) mild histopathologic changes/mild presence of immunolabelled cells; (3) moderate histopathologic changes/moderate presence of immunolabelled cells; (4) severe histopathologic changes/abundant presence of immunolabelled cells. Morphological features including cell size, as well as anatomical location were the criteria applied to tentatively identify the types of cells that were immunolabelled.

### Statistical analysis

Statistical analyses and data visualisation were performed with GraphPad Prism version 9 (GraphPad Software La Jolla, CA, USA). The differences in total scores between the experimental groups with respect to histopathological changes and cells immunolabelled for viral antigen, as well as the differences between the experimental groups in the various organs evaluated, were tested for statistical significance using an unpaired *t*-test.

## Results

### Trends in the evolution of virus antigen and histopathologic lesion scores over time

**Figure 1:**
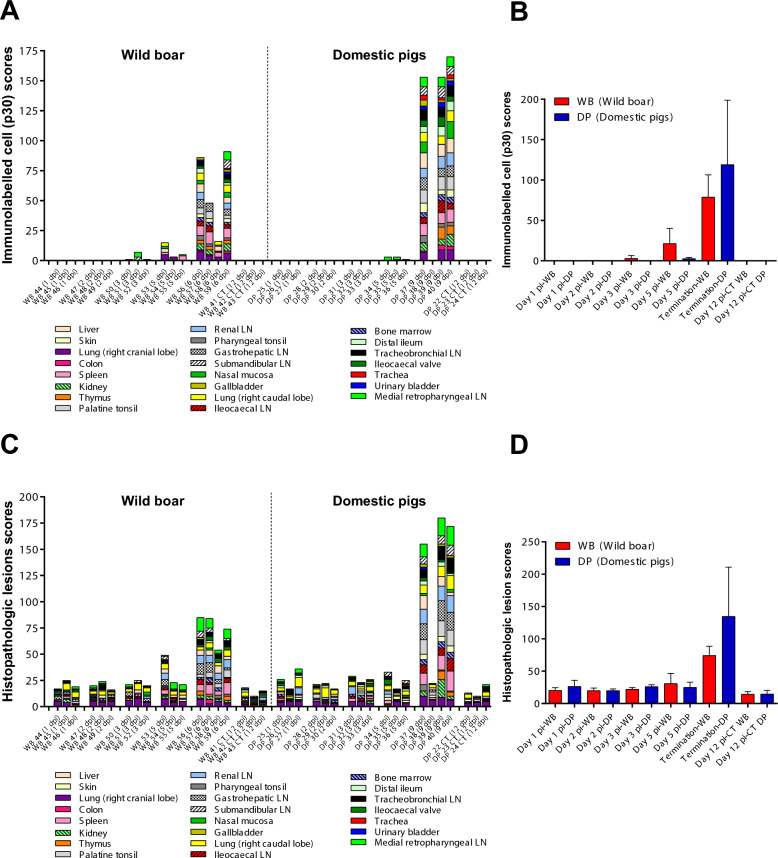
**Trends in the evolution of virus antigen and histopathologic change scores in wild boar (*Sus scrofa scrofa*) and domestic pigs (*Sus scrofa domesticus*) intranasally inoculated with African Swine Fever Virus genotype II “Armenia 2007” strain.** Virus antigen (**A**) and histopathologic changes (**C**) were scored and documented per individual. A semiquantitative assessment of cells immunolabelled for viral antigen (protein p30/CP204L) and histopathological changes severity was performed in different histological structures of the organs, which were evaluated as follows: (0) no presence of immunolabelled cells/no histopathologic changes; (1) occasional presence of immunolabelled cells/minimal histopathologic changes; (2) mild presence of immunolabelled cells/mild histopathologic changes; (3) moderate presence of immunolabelled cells/moderate histopathologic changes; (4) abundant presence of immunolabelled cells/severe histopathologic changes. Each multicoloured bar represents the cumulative score observed in the different organs of the examined animals and the Immunolabelled cell/histopathologic scores for each organ are represented within the bars as different colours. Immunolabelled cell/histopathologic scores are shown on the y-axis and individual animals evaluated in each experimental group and euthanised on different dates after infection are shown on the x-axis. Uninfected animals (CT) euthanised on 12 dpi are also shown. (**B**, **D**) Mean ± SD of the cumulative virus antigen/histopathologic change scores (y-axis) in each group of pigs or wild boar euthanised on different days after infection (x-axis). Uninfected animals (CT) euthanised on 12 dpi are also shown (x-axis). Statistical analysis was performed using an unpaired t-test. No significant differences were observed.

Immunohistochemical evaluations detected cells immunolabelled against ASF-p30 antigen as early as 3 dpi in wild boar tissues (Figure 1A), whereas in pigs, the viral antigen was not detected until 5 dpi (Figure 1A). Wild boar showed higher virus antigen scores than pigs at 5 dpi (Figure 1B). At the clinical endpoint, virus antigen scores were higher in pigs compared to wild boar, but not statistically significant (Figure 1B; *p* = 0.37). A single pig DP38 was euthanised at 9 dpi to avoid single housing following euthanasia of its three pen mates that had reached humane endpoint. No immunolabelled cells or significant histopathologic alterations were observed in any of the organs in pig DP38.

Histopathological lesion scores of inoculated pigs and wild boar were virtually the same from 1 to 3 dpi, showing a variety of mild and non-specific findings similar to those observed in control animals (Figure 1C). These consisted mainly of interstitial and alveolar oedema in the lungs, together with mild hyperaemia and occasional small haemorrhages observed in the medulla of the lymph nodes (gastrohepatic, tracheobronchial, medial retropharyngeal and renal), which in the absence of viral antigen, could be attributed to the euthanasia procedure. At 5 dpi, wild boar exhibited a slightly higher, albeit non-significant, histopathological score compared to pigs (Figure 1D) and pathological changes were associated with the presence of viral antigen in wild boar. Only the groups of animals that were euthanised at the humane endpoint showed a marked increase in histopathological scores compared to the uninfected control groups. Scores were substantially higher, but not significantly (Figure 1d; *p* = 0.16) in pigs (euthanised at 9 dpi) compared to wild boar (euthanised at 6 dpi). In summary, the number of organs with histopathological lesions and the severity of these lesions increased as the experiment progressed. Lesions became apparent following viral replication and coincided with antigen detection.

### Dynamics of viral antigen distribution and appearance of histopathological lesions

**Figure 2:**
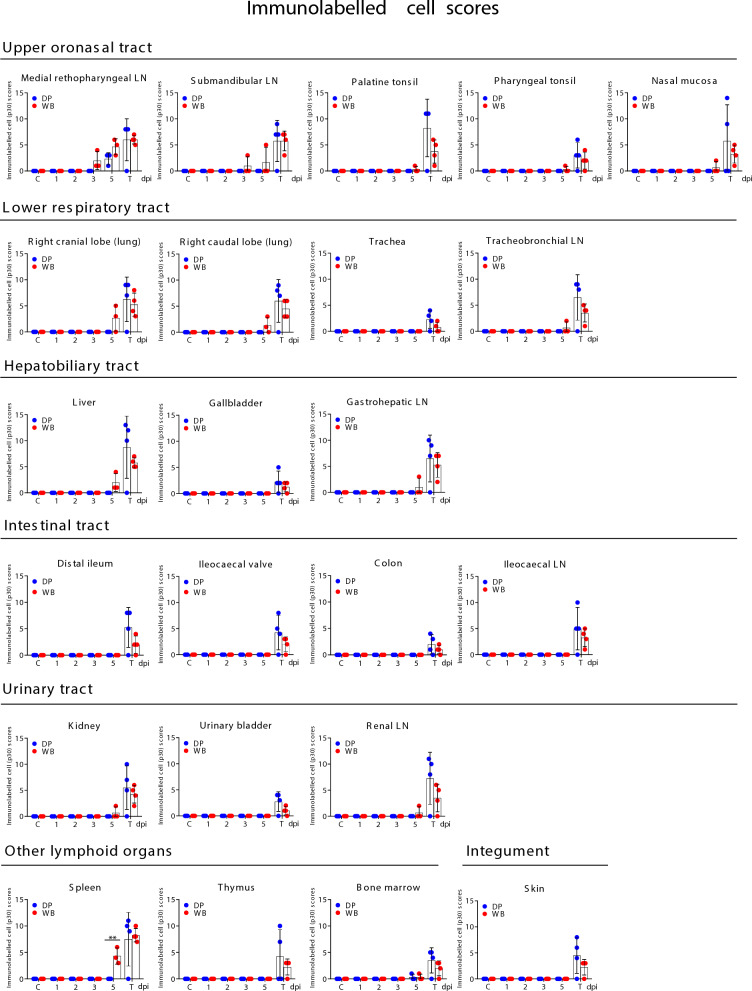
**Comparative evaluation of the dynamics of virus antigen scores (mean ± SD) in each of the examined organs in wild boar (*Sus scrofa scrofa*) and domestic pigs (*Sus scrofa domesticus*) intranasally inoculated with African Swine Fever Virus genotype II “Armenia 2007” strain.** Each animal is represented by a dot within its respective group: Domestic pigs (DP); Wild boar (WB). Statistical analysis was performed using an unpaired t-test. Black asterisks indicate statistically significant differences between the two groups of infected animals (wild boar and domestic pigs) euthanised on different dates; significant variables (*P ≤ 0.05; **P ≤ 0.01; ***P ≤ 0.001). Score of immunolabelled cells (y-axis); day-post infection (dpi); uninfected control groups (C); lymph node (LN); groups of infected animals euthanised at humane endpoint (T): 6 dpi for WB and 9 dpi for DP.

**Figure 3:**
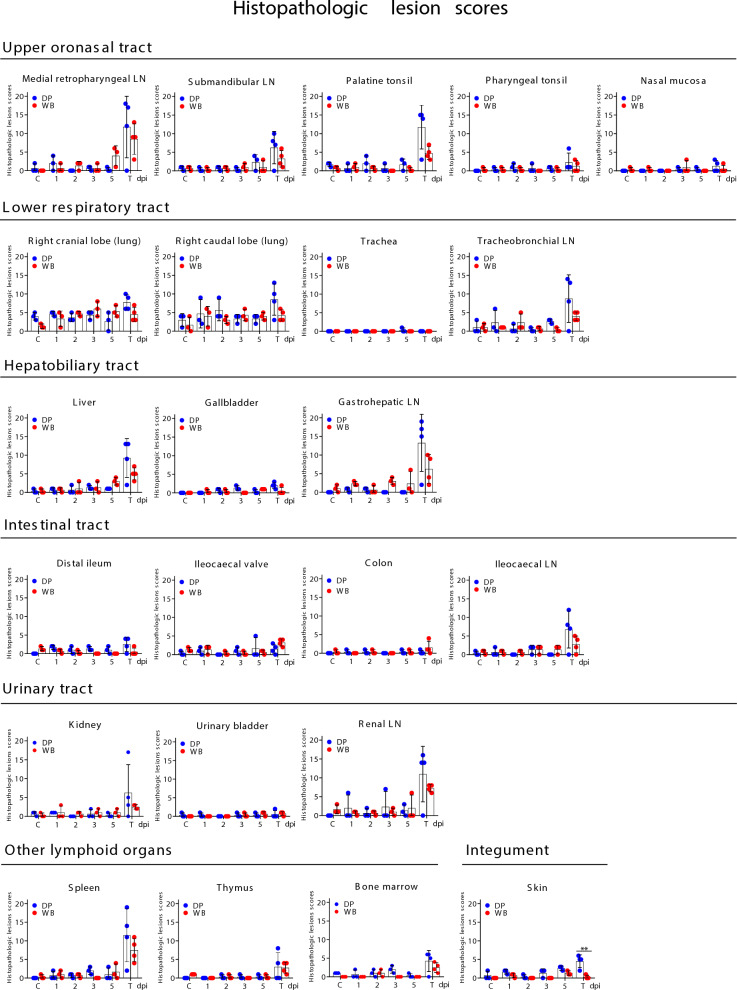
**Comparative evaluation of the dynamics of histopathological change scores (mean ± SD) in each of the examined organs in wild boar (*Sus scrofa scrofa*) and domestic pigs (*Sus scrofa domesticus*) intranasally inoculated with African Swine Fever Virus genotype II “Armenia 2007” strain.** Each animal is represented by a dot within its respective group: domestic pigs (DP); wild boar (WB). Statistical analysis was performed using an unpaired *t*-test. Black asterisks indicate statistically significant differences between the two groups of infected animals (wild boar and domestic pigs) euthanised on different dates; significant variables (*P ≤ 0.05; **P ≤ 0.01; ***P ≤ 0.001). Score of immunolabelled cells (y-axis); day-post infection (dpi); uninfected control groups (C); lymph node (LN); groups of infected animals euthanised at humane endpoint (T): 6 dpi for WB and 9 dpi for DP.

The dynamics of the virus antigen and the histopathologic change scores in each of the organs evaluated are plotted over time in Figures 2 and 3. The total scores for immunolabelled cells and histopathological lesions are presented for each individual (Additional file 1).

#### Early stage of infection (1–5 dpi)

**Figure 4:**
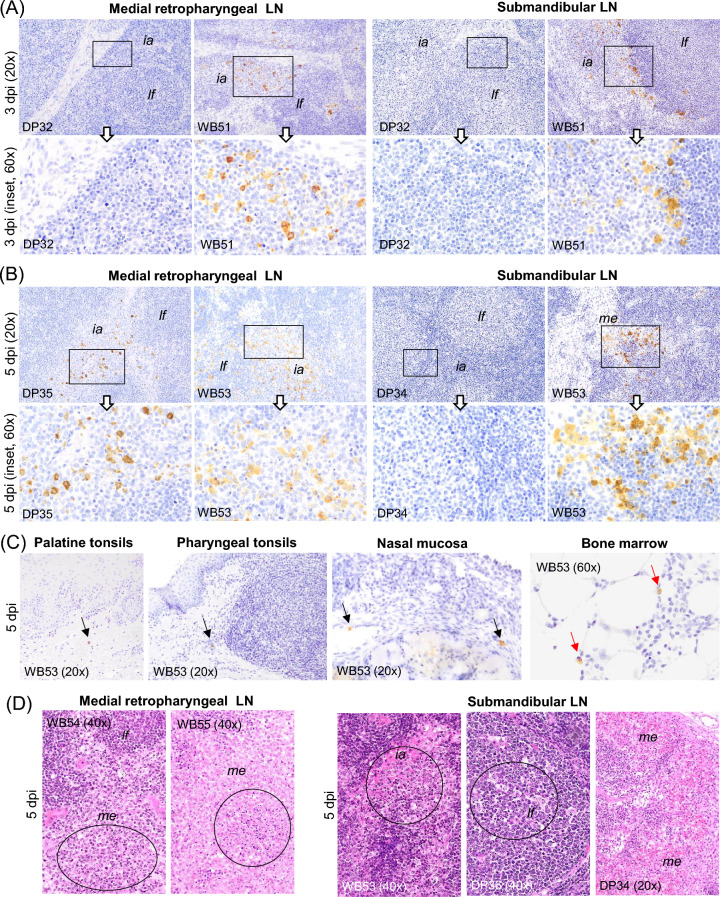
**Histopathologic changes and presence of cells immunolabelled for ASFV-specific antigen (P30).** Organs taken from wild boar (*Sus scrofa scrofa*) and domestic pigs (*Sus scrofa domesticus*) intranasally inoculated with African Swine Fever Virus genotype II "Armenia 2007" strain euthanised between 1 and 5 dpi. (**A**, **B**) IHC. Representative images of cells, primarily macrophages, immunolabelled for viral antigen in the medial retropharyngeal (MRPLN) and submandibular lymph nodes at 3 and 5 dpi. **A** Note that viral antigen was only present in the WB at 3 dpi. **B** MRPLN was also the first organ in which viral antigen was detected in the DP at 5 dpi. **C** IHC. Intravascular monocytes and infiltrating macrophages immunolabelled for the virus in tonsils (palatine and pharyngeal) and nasal mucosa (black arrows) at 5 dpi. Immunolabelled myeloid cells (red arrows) were also observed in the bone marrow of a wild boar at 5 dpi. **D** HE staining. Characteristic pyknotic and fragmented cell nuclei (karyorrhexis, indicated by circles) observed in the medulla and interfollicular areas of the MRPLN in wild boar. Karyorrhexis (circles) was also observed in the medulla, interfollicular areas and lymphoid follicles of the submandibular lymph node in wild boar and pigs, together with multifocal haemorrhages in the medulla of DP34. Immunohistochemistry against P30 protein (IHC); Haematoxylin–eosin staining (HE); Original magnification (number x); interfollicular areas (*ia*); lymphoid follicle (*lf*); medullary area (*me*); Wild boar (WB); Domestic pig (DP); White arrows indicate histopathological details of the selected areas within the boxes at higher magnification.

The medial retropharyngeal lymph node (MRPLN), located in the oronasal tract, was the first organ in which cells immunolabelled against ASFV-specific antigen were detected. This occurred at 3 dpi in all three wild boar and 5 dpi for the three pigs (Figure 2). Immunolabelled cells morphologically consistent with macrophages were mainly observed in the medullary area, but in wild boar WB51, these cells were also observed in the interfollicular areas and occasionally within lymphoid follicles (Figure 4A). Additionally at 3 dpi, wild boar WB51 also exhibited immunolabelling in the submandibular lymph node, with virus antigen detected in macrophages within the medullary and interfollicular areas. In comparison, none of the pigs euthanised at 3 dpi showed any immunolabelled cells anywhere (Figure 4A).

At 5 dpi, in addition to the MRPLN in all three wild boar, viral antigen was detected in the other organs of the oronasal tract (Figure 2). Wild boar WB53 showed immunolabelled macrophages in the medullary and interfollicular areas of the submandibular LN (Figure 4B), immunolabelled intravascular monocytes i.e. -large in size with abundant cytoplasm and an indented or bean-shaped nucleus—in the palatine tonsil, occasional immunolabelled macrophages in the lamina propria of the pharyngeal tonsil as well as intravascular immunolabelled monocytes and macrophages infiltrating the lamina propria of the nasal mucosa (Figure 4C, black arrows). However, the MRPLN was the only organ in the oronasal tract that showed immunolabelled cells in the three pigs euthanised at 5 dpi (Figures 2 and 4B). The bone marrow was the only other location, and only in pig DP35, where some immunolabelled cells (myeloid cells) were observed in pigs culled at 5 dpi. The same immunostaining pattern was also observed in the bone marrow of wild boar WB53 at 5 dpi (Figure 4C, red arrows). The bone marrow showed no histopathological alterations in either animal.

No conspicuous histopathological changes were observed in any of the evaluated organs of the oronasal tract until 5 dpi (Figure 3), when mild to moderate alterations such as pyknotic and fragmented cell nuclei (karyorrhexis) were observed in medullary and interfollicular areas of the MRPLN of wild boar WB54 and WB55 (Figure 4D). In addition, mild karyorrhexis was observed in the medullary, interfollicular areas and lymphoid follicles of the submandibular LN in wild boar WB53 and pig DP36, despite the absence of viral antigen in the latter, as well as moderate, multifocal haemorrhages in the medulla of pig DP34 (Figure 4D). On 5 dpi, no conspicuous lesions were observed in the palatine tonsils, pharyngeal tonsils or nasal mucosa in either pigs or wild boar.

Viral antigen was also detected in other tissues at 5 dpi in some wild boar, but in none of the pigs (Figure 2). These included the lower respiratory tract (right cranial and caudal lung lobes and tracheobronchial LN), hepatobiliary tract (liver and gastrohepatic LN), urinary tract (kidney and renal LN) and other lymphoid organs (spleen). No viral antigen was detected in any of the other organs evaluated up to 5 dpi in the wild boar.

**Figure 5:**
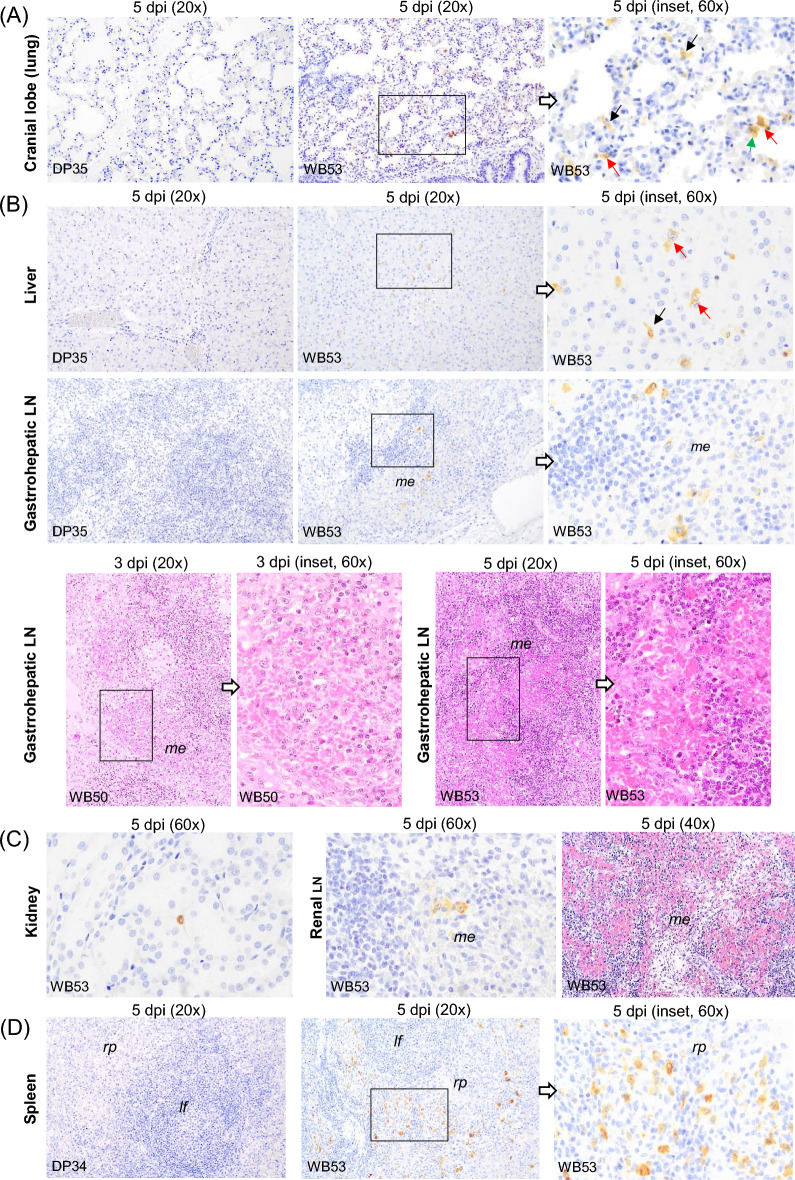
**Histopathologic changes and presence of cells immunolabelled for ASFV-specific antigen (P30)**. Organs taken from wild boar (*Sus scrofa scrofa*) and domestic pigs (*Sus scrofa domesticus*) intranasally inoculated with African Swine Fever Virus genotype II ‘Armenia 2007’ strain euthanised between 1 and 5 dpi. **A** Right cranial lung lobe, IHC. At 5 dpi, immunolabelled cells were only present in wild boar. Note the presence of immunolabelled interstitial macrophages (black arrows) and alveolar macrophages (green arrow). Immunolabelled pneumocytes were also occasionally observed (red arrows). **B** Liver and gastrohepatic lymph node, IHC. At 5 dpi, immunolabelled cells were visible only in wild boar. Note the presence of immunolabelled hepatocytes (red arrows) and Kupffer cells (black arrows) in the liver, as well as the occasional presence of immunolabelled macrophages in the medullary areas of the gastrohepatic lymph node; Gastrohepatic lymph node, HE staining. Medullary haemorrhages (WB50, 3 dpi); Haemorrhages affecting medullary areas (WB53, 5 dpi). **C** Kidney, IHC. Immunolabelled interstitial macrophage (WB53, 5 dpi). In the same animal, the renal lymph node showed occasional immunolabelled cells (IHC) as well as diffuse haemorrhages (HE staining) in the medullary and interfollicular areas. **D** Spleen, IHC, 5 dpi. Immunolabelled macrophages were present in the spleens of the three wild boars euthanised at 5 dpi, mainly in the red pulp, but not in any of the pigs euthanised on the same day. Immunohistochemistry against P30 protein (IHC); Haematoxylin–eosin staining (HE); Original magnification (number x); interfollicular areas (*ia*); lymphoid follicle (*lf*); medullary area (*me*); splenic red pulp *(rp)*; Wild boar (WB); Domestic pig (DP); White arrows indicate histopathological details of the selected areas within the boxes at higher magnification.

In the lung, there was a moderate amount of immunolabelled interstitial macrophages (Figure 5A, black arrows) and some alveolar macrophages (Figure 5A, green arrow) in the cranial lung lobe of wild boar WB53 and WB54. In wild boar WB53, immunolabelled cells morphologically consistent with type II pneumocytes were also occasionally observed (Figure 5A, red arrows). In comparison, there were fewer immunolabelled cells in the caudal lung lobe, and antigen was only detected in macrophages. Occasionally, immunolabelled macrophages were also found in the interfollicular areas and within the lymphoid follicles in the tracheobronchial LN of wild boar WB53. Up to 5 dpi, both pigs and wild boar only showed nonspecific lung lesions similar to those described in the control groups, such as moderate, diffuse congestion, moderate interstitial and alveolar oedema and mild peribronchial mononuclear infiltrates. Moderate to severe medullary haemorrhages observed in the tracheobronchial LN of pig DP26 on 1 dpi and in wild boar WB48 on 2 dpi were considered to be nonspecific lesions or artefacts caused by tissue processing.

All three wild boar euthanised at 5 dpi showed intravascular immunolabelled monocytes within the liver (Figure 2). These were observed mainly in the sinusoids, but also in the portal and central veins of WB53, in which immunolabelled hepatocytes (Figure 5B, red arrows) and Kupffer cells (Figure 5B, black arrow) also could be detected. In this animal, a small number of immunolabelled macrophages were also seen in the gastrohepatic LN, in the medullary areas and occasionally in the interfollicular areas (Figure 5B). However, while no conspicuous histopathological findings were observed in the livers up to 5 dpi, the gastrohepatic LN in wild boar WB50 euthanised at 3 dpi showed moderate medullary haemorrhages (Figure 5B). Haemorrhages could also be observed affecting medullary and interfollicular areas in wild boar WB53 euthanised at 5 dpi (Figure 5B).

In the urinary tract, wild boar WB53 was the first animal to show occasional immunolabelled mononuclear interstitial infiltrates (Figure 5C) and intravascular monocytes in the kidney at 5 dpi. In addition, the renal LN of this animal showed occasional immunolabelled cells in the medullary and interfollicular areas (Figure 5C). However, while kidneys from wild boar and pigs euthanised up to 5 dpi showed no histopathological alterations, severe, diffuse haemorrhages involving the medullary and interfollicular areas were observed in the renal LN of pig DP31 at 3 dpi and in wild boar WB53 at 5 dpi (Figure 5C).

As for other lymphoid organs, immunolabelled macrophages were detected in the spleen of the three wild boars euthanised at 5 dpi, but not in any of the pigs euthanised on the same dpi (significant differences in viral antigen score, *p* = 0.008). These macrophages were mainly found in the red pulp (Figure 5D), with occasional viral antigen also observed in the ellipsoids. In wild boar, immunolabelled macrophages were also occasionally detected within the lymphoid follicles of WB54 and WB55. The spleen of both pigs and wild boar euthanised up to 5 dpi showed no histopathological changes that differed from those observed in the spleen of control animals.

#### Humane endpoints (6 dpi for wild boar and 9 dpi for pigs)

One pig, DP38, did not reach the humane endpoint but was euthanised to avoid anxiety and stress associated with single housing. No viral antigen or histopathological changes were observed in this animal; therefore, it is not included in the results below. For all other pigs and wild boar at the humane endpoint, viral antigen and histopathological lesions were widespread in many organ systems.

**Figure 6:**
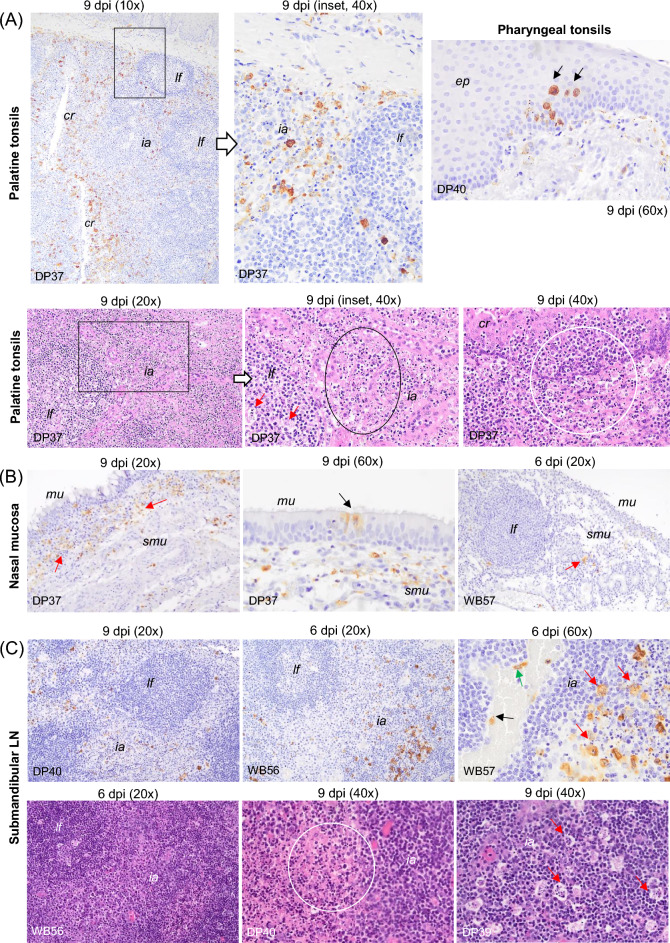
**Histopathologic changes and presence of cells immunolabelled for ASFV-specific antigen (P30).** Organs taken from infected animals euthanised at humane endpoint (6 dpi for wild boar and 9 dpi for pigs). **A** Palatine and pharyngeal tonsil, IHC. Cells immunolabelled for viral antigen, mainly macrophages and occasional lymphocytes, infiltrating the crypt epithelium, the diffuse lymphoid tissue surrounding the crypts, the interfollicular areas and the lamina propria. Observe the scarcity of viral antigen within the lymphoid follicles. The pharyngeal tonsil also showed immunolabelled epithelial cells in the epithelium (black arrows); Palatine tonsil, HE staining. Lymphoid depletion in the interfollicular areas, cell fragmentation (oval circle) and tingible body macrophages within the lymphoid follicles (red arrows). Note the infiltrates of mononuclear cells displaying severe pyknosis and cell fragmentation in the crypt epithelium (white circle). **B** Nasal mucosa, IHC. Immunolabelled macrophages in mucosa and submucosa. Note the occasional presence of immunolabelled epithelial cells in the respiratory epithelium (black arrow). Occasional immunolabelled macrophages were also observed in the mucosa and interfollicular areas of the nasal mucosa in wild boar (red arrow). **C** Submandibular lymph node, IHC. Marked presence of immunolabelled macrophages in the medullary and interfollicular areas. Note the presence of phagocytised immunolabelled cell debris (tingible bodies, red arrows) as well as intravascular monocytes (black arrow) and endothelial cells (green arrow) immunostained; HE staining. Areas with lymphoid depletion accompanied by abundant pyknotic cells, karyorrhexis (circle) and macrophages showing abundant cytoplasm containing phagocytised cell debris (tingible body macrophages, red arrows). Immunohistochemistry against P30 protein (IHC); Haematoxylin–eosin staining (HE); Original magnification (number x); interfollicular areas (*ia*); lymphoid follicle (*lf*); epithelium (*ep*); crypt epithelium (*cr*); mucosa (*mu*), submucosa (*smu*); Wild boar (WB); Domestic pig (DP); White arrows indicate histopathological details of the selected areas within the boxes at higher magnification.

**Figure 7:**
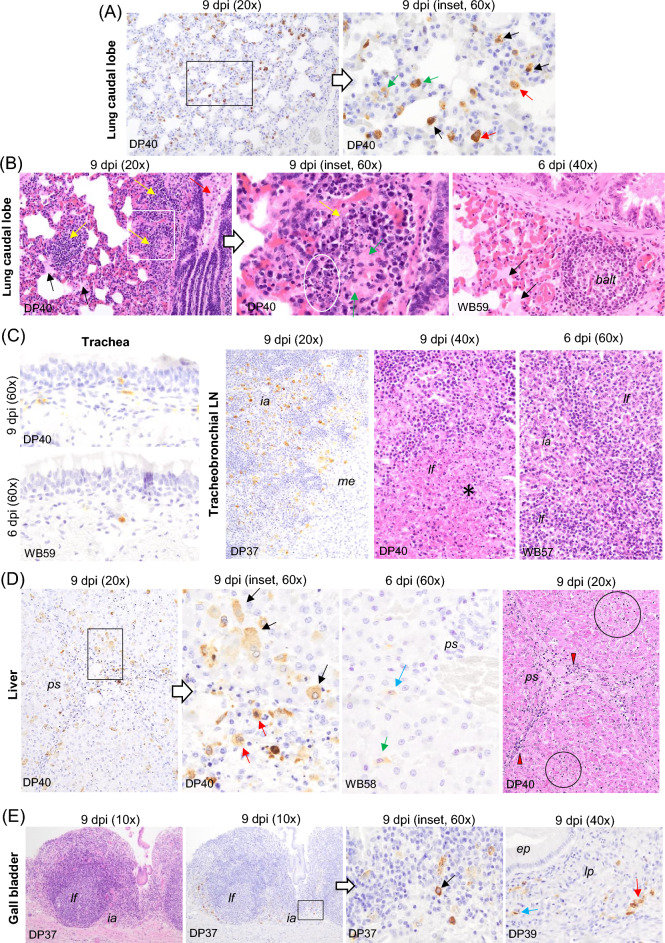
**Histopathologic changes and presence of cells immunolabelled for ASFV-specific antigen (P30).** Organs taken from infected animals euthanised at humane endpoint (6 dpi for wild boar and 9 dpi for pigs). **A** Lung caudal lobe, IHC. Abundant Interstitial (green arrows), intravascular (black arrows) and alveolar (red arrows) macrophages immunolabelled; **B** Lung caudal lobe, HE staining. Alveolar septal thickening along with mononuclear cell infiltrates consisting mainly of macrophages and lymphocytes (yellow arrows), oedema (black arrows), cell debris (white circle) and fibrin deposits (green arrows) in the alveolar lumen. Note also the presence of inflammatory cells in the bronchiolar lumen (bronchiolitis, red arrow); **C** Trachea and tracheobronchial lymph node, IHC. Macrophages infiltrating the mucosa and the epithelium of the trachea; Tracheobronchial lymph node, IHC. Immunolabelled macrophages in the medullary and interfollicular areas; HE staining. Note the presence of karyorrhexis and haemorrhages (asterisk) within lymphoid follicles of pigs compared to mild lymphoid tissue depletion in wild boar; **D** Liver, IHC. Abundant immunolabelled hepatocytes (black arrows) and interstitial macrophages in the portal spaces (red arrows). Occasional stained Kupffer cells (green arrow) and intravascular monocytes (blue arrows) were also observed; HE staining. Liver damage was characterised by the presence of interstitial mononuclear infiltrates in the portal spaces and interlobular septa areas, which exhibited karyorrhexis (red arrowheads), and multifocal necrotic foci of hepatocytes with mononuclear cell infiltration (circles); **E** Gallbladder, IHC. Occasional immunolabelled macrophages in the interfollicular areas of the lymphoid tissue (black arrow) and the lamina propria (red arrow). Occasionally immunolabelled endothelial cells (blue arrow). Immunohistochemistry against P30 protein (IHC); Haematoxylin–eosin staining (HE); Original magnification (number x); interfollicular areas (*ia*); lymphoid follicle (*lf*); medullary area (*me*), epithelium (*ep*); lamina propria (*lp*), portal space (*ps*); bronchus-associated lymphoid tissue (*balt*); Wild boar (WB); Domestic pig (DP); White arrows indicate histopathological details of the selected areas within the boxes at higher magnification.

**Figure 8:**
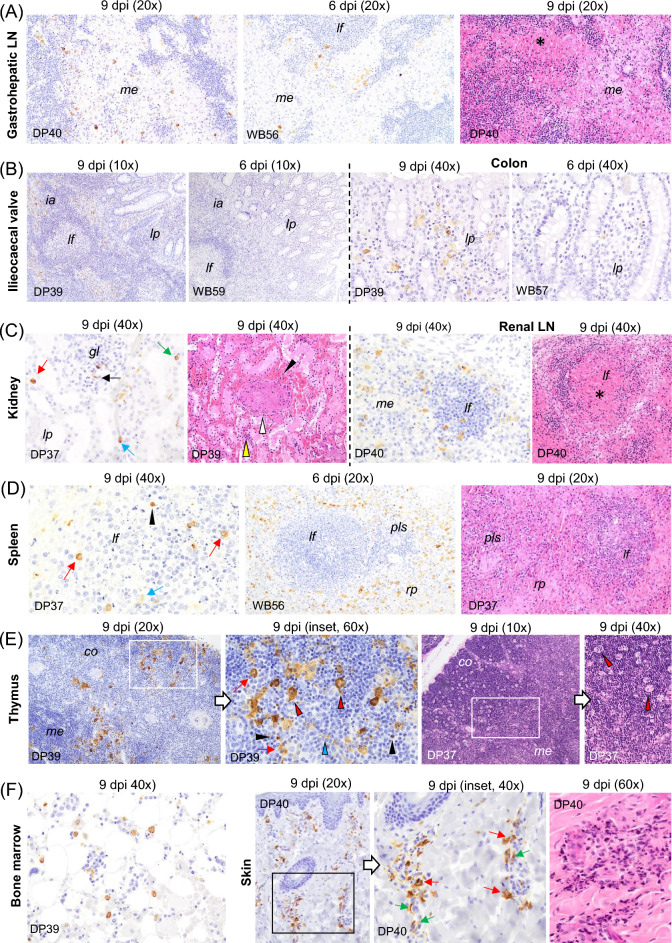
**Histopathologic changes and presence of cells immunolabelled for ASFV-specific antigen (P30).** Organs from infected animals euthanised at humane endpoint (6 dpi for wild boar and 9 dpi for pigs). **A** Gastrohepatic lymph node, IHC. Immunolabelled macrophages in medulla and interfollicular areas; HE staining. Haemorrhages (asterisk) in medullary areas; **B** Ileocaecal valve and colon, IHC. Interfollicular areas and lamina propria with immunolabelled macrophages in pigs. In wild boar immunostained cells were scarce; **C** Kidney, IHC. Capillary endothelial cells within glomeruli (black arrow), interstitial macrophages (red arrow), intravascular monocytes (green arrow), and epithelial cells of the renal ducts (blue arrow) immunolabelled; HE staining. Haemorrhages cortex (black arrowhead), vasculitis and microthrombi (white arrowhead). Tubular nephrosis and interstitial mononuclear infiltrates also observed (yellow arrowhead); Renal LN, IHC and HE staining. Immunolabelled macrophages in medullary and interfollicular areas. Haemorrhages (asterisk) and lymphoid depletion in lymphoid follicle; **D** Spleen, IHC. Abundant immunolabelled macrophages in red pulp with occasional presence within lymphoid follicles (red arrows). Immunolabelled lymphocytes (black arrowhead) and endothelial cells (blue arrow) also observed; HE staining. Lymphoid depletion affecting lymphoid follicles and periarteriolar sheaths; **E** Thymus, IHC. Macrophages (red arrows), tingible body macrophages (red arrowheads) and occasional lymphocytes (black arrowheads) immunolabelled in cortex and medulla. Occasionally stellate cells consistent with reticuloepithelial cells (blue arrowhead); HE staining. Lymphoid depletion, Karyorrhexis and tingible body macrophages in cortex (red arrowheads); **F** Bone marrow, IHC. Immunostained cells, mainly myeloid cells; Skin, IHC. Perivascular mononuclear infiltrates with numerous immunolabelled macrophages in pigs (red arrows) and Capillary endothelial cells (green arrows); HE staining. Perivascular mononuclear infiltrates and vasculitis. Immunohistochemistry against P30 protein (IHC); Haematoxylin–eosin staining (HE); Original magnification (number x); interfollicular areas (*ia*); lymphoid follicle (*lf*); medullary area (*me*); lamina propria (*lp*); glomeruli (*gl*), red pulp (*rp*), periarteriolar lymphoid sheaths (*ps*), cortex (*co*); Wild boar (WB); Domestic pig (DP).

A common pattern was seen in the lymph nodes (medial retropharyngeal, submandibular, tracheobronchial, gastrohepatic, ileocaecal and renal) assessed in each of the organ tracts in the four boars, as well as in the three pigs (DP37, DP39 and DP40) that reached the humane endpoint. All of them showed a large number of macrophages immunolabelled for viral antigen. These were observed mainly in the medullary and interfollicular areas, but also occasionally within the lymphoid follicles. Many of these macrophages had an increased amount of cytoplasm containing phagocytised, immunolabelled cell debris (tingible bodies; regarded to represent remains of phagocytised apoptotic cells). In addition, immunolabelled intravascular monocytes, endothelial cells and reticular cells were occasionally observed (Figures 6C, 7C and 8A, C). Viral antigen scores were usually higher in pig than in wild boar (Figure 2, Additional file 1). Multifocal and diffuse haemorrhages in the medulla and interfollicular areas, together with mild to focally severe lymphoid depletion, were the most notable histopathological lesions in these lymph nodes in both pigs and wild boar. This was accompanied by features of cell death (karyorrhexis) in the interfollicular areas and lymphoid follicles. The overall severity was slightly lower in wild boar (Figures 6C, 7C and 8A, C). Among pigs, the lesions were particularly severe in DP39 and DP40, while WB56 showed the most prominent changes among wild boar.

In the pharyngeal and palatine tonsils, immunolabelled cells could be seen in the three pigs that reached the humane endpoint at 9 dpi (pig DP37, DP39, and DP40) (Figure 2). These cells were mainly macrophages and occasional lymphocytes that infiltrated epithelium of the crypts, the diffuse lymphoid tissue surrounding the crypts, the interfollicular areas and the lamina propria. Antigen-positive cells within the lymphoid follicles were scarce (Figure 6A). Pig DP40 also had immunolabelled epithelial cells in the epithelium of the pharyngeal tonsil (Figure 6A, black arrows). Wild boar euthanised at the humane endpoint (6 dpi) showed a similar pattern in both tonsils, but with lower virus antigen scores (Figure 2). Mild lymphoid depletion, together with moderate amount of pyknotic cells and cell fragmentation in interfollicular areas and lymphoid follicles, were the most obvious histopathological lesions in the pharyngeal tonsil of pig DP37. In contrast, the palatine tonsil of pigs DP37, DP39 and DP40 showed severe lymphoid depletion in the interfollicular areas and mild lymphoid depletion in the lymphoid follicles, together with cell fragmentation (Figure 6A, HE staining, black circle) and tingible body macrophages. The latter were especially abundant within the lymphoid follicles (Figure 6A, red arrows). The epithelium of the crypts also showed moderate to severe infiltrates of mononuclear cells displaying severe pyknosis and cell fragmentation (Figure 6A, HE staining, white circle). These lesions, although less severe, were also present in wild boar at the humane endpoint. Like the tonsils, the nasal mucosa did not begin to show evidence of viral antigen in pigs until 9 dpi, with large numbers of immunolabelled macrophages mainly in the mucosa, submucosa (Figure 6B, red arrows) and perichondrium. Occasionally, immunolabelled epithelial cells could also be seen in the respiratory epithelium (Figure 6B, black arrow) along with occasional labelled endothelial cells (Figure 6B, green arrows). The wild boar group euthanised at 6 dpi showed a scant number of immunolabelled macrophages in the mucosa and interfollicular areas of the nasal mucosa (Figure 6B, red arrows). Apart from mild karyorrhexis in lymphoid tissue in pig DP37, pig DP40 and wild boar WB57, no other specific histopathological lesions were observed in the nasal mucosa.

In the lower respiratory tract of pigs DP37, DP39 and DP40, there was an abundance of interstitial (Figure 7A, green arrows) and intravascular (Figure 7A, black arrows) immunolabelled macrophages in both the cranial and caudal lobes, as well as fewer immunolabelled alveolar macrophages (Figure 7A, red arrows) and occasional immunolabelled pneumocytes. Additionally, numerous macrophages within the bronchial-associated lymphoid tissue (BALT) were immunolabelled in pig DP39. All four wild boar showed a similar immunostaining pattern, but without the presence of immunolabelled intra-BALT macrophages. Histopathological evaluation of pigs revealed moderate alveolar septal thickening in the lungs, along with mild to moderate mononuclear cell infiltrates consisting mainly of macrophages and lymphocytes (Figure 7B, yellow arrows), oedema (Figure 7B, black arrows), cell debris (Figure 7B, white circle) and fibrin deposits (Figure 7B, green arrows) in the alveolar lumen. This was accompanied by bronchiolar oedema, sloughed epithelium and mononuclear cells (bronchiolitis; Figure 7B, red arrows), vascular hypertrophy as well as pyknotic cells and karyorrhexis in the BALT. The latter finding was particularly notable in the caudal lobe. However, in wild boar the lesions were less severe, with only diffuse, mild congestion and alveolar oedema (Figure 7B, WB59) together with minimal vascular endothelial hypertrophy.

In the trachea, all three pigs and two of the four wild boar, WB57 and WB59, that reached the humane endpoint showed immunolabelled cells. These consisted mainly of macrophages infiltrating the mucosa and occasionally the epithelium (Figure 7C). In the trachea, no lesions were found throughout the experiment, neither in pigs nor wild boar.

In the case of pigs, viral antigen was detected in organs of the hepatobiliary tract only in the animals that reached the humane endpoint (Figure 2). Their livers displayed a large number of hepatocytes (Figure 7D, black arrows) and interstitial macrophages in the portal spaces (Figure 7D, red arrows) immunolabelled for viral antigen. Immunolabelled Kupffer cells (Figure 7D, green arrow) and intravascular monocytes (Figure 7D, blue arrow) were also present. In wild boar, the same type of cells were immunolabelled, but they were less abundant. Extensive liver damage was also evident in the euthanised pigs at the humane endpoint. Frequently centred in the portal areas or interlobular septa, the lesions were characterised by mild to moderate interstitial mononuclear infiltrates that exhibited karyorrhexis (Figure 7D, HE staining, red arrowheads). In addition, foci of hypereosinophilic hepatocytes with nuclear karyorrhexis and karyolysis (necrosis) accompanied by mononuclear cell infiltration were observed (Figure 7D, HE staining, black circles). An increase in intravascular leukocytes in the hepatic sinusoids (sinusoidal leukocytosis) was also noted, along with the presence of enlarged Kupffer cells. These findings were more prominent in pigs DP37 and DP40. In comparison, hepatic lesions were limited to diffuse, mild to moderate liver congestion in all four wild boar, along with mild leucocytosis only observed in wild boar WB58.

In the gallbladder, Pig DP37 showed a moderate amount of immunolabelled macrophages in the interfollicular areas of the lymphoid tissue of the submucosa (Figure 7E, black arrow). Occasional macrophages in the lamina propria (Figure 7E, red arrow) and intravascular monocytes were also seen in pig DP37, DP39 and DP40. Positive intravascular monocytes could also be found in wild boar WB56, WB57 and WB59. Pig DP40 and wild boar WB56 and WB59 also showed occasionally immunolabelled endothelial cells (Figure 7E, blue arrow). Histological examination revealed minimal to mild mononuclear infiltrates in the lamina propria of the gallbladder of pigs DP37, DP39, DP40 and wild boar WB56, with wild boar WB57 also showing mild, submucosal oedema.

In the distal ileum, ileocaecal valve and the colon of the three pigs that reached the humane endpoint, there were abundant immunolabelled macrophages, most frequently observed in the interfollicular areas, followed by the lamina propria. In contrast immunolabelled macrophages were only occasionally observed in the lamina propria in wild boar (Figure 8B). Microscopically, minimal lesions restricted to the lamina propria and characterised by the presence of mild hyperaemia and mild mononuclear infiltrate were observed in the distal ileum, ileocaecal valve and colon only in some pigs and wild boar.

In the urinary tract of all three pigs, immunolabelled cells were mainly observed in the renal cortex. These were identified as capillary endothelial-like cells within the glomeruli (Figure 8C, black arrow), interstitial macrophages (Figure 8C, red arrow), intravascular monocytes (Figure 8C, green arrow), and epithelial cells of the renal ducts (Figure 8C, blue arrow). There were fewer labelled cells in the medulla and pelvic area. In all four wild boar, the same type of cells were immunolabelled and evenly distributed throughout the kidney but observed less frequently. Histopathological changes of the kidney were mild in all four wild boar as well as in pigs DP37 and DP40, which showed mild diffuse congestion and occasional small, interstitial mononuclear infiltrates in the renal cortex. The exception was pig DP39, which showed severe, diffuse congestion together with severe, multifocal haemorrhages in the renal cortex (Figure 8C, HE staining, black arrowhead), medulla and pelvic area, accompanied by vasculitis and microthrombi mainly in the cortex (Figure 8C, HE staining, white arrowhead). In addition to moderate interstitial mononuclear infiltrates with pyknotic cells and moderate karyorrhexis, tubular nephrosis was also observed in the renal cortex in this animal (Figure 8C, HE staining, yellow arrowhead). In the urinary bladder, small numbers of immunolabelled macrophages in the lamina propria and occasional endothelial cells were observed in all animals reaching the humane endpoint except for wild boar WB58. However, no specific lesions were observed in any of the animals throughout the experiment.

Regarding other lymphoid organs, which are considered main targets for ASFV, the spleen contained abundant immunolabelled macrophages. These were observed mainly in the red pulp, but also occasionally in lymphoid follicles (Figure 8D, red arrows), periarteriolar lymphoid sheaths and ellipsoids in both the pigs and the wild boar that reached the humane endpoint. In pig DP37, there were also occasional immunolabelled lymphocytes (Figure 8D, black arrowhead) and endothelial cells (Figure 8D, blue arrow). In pigs, histopathological changes included moderate to severe karyorrhexis in the red pulp, lymphoid follicles and periarteriolar sheaths, accompanied by mild to severe lymphoid depletion of the white pulp (Figure 8D, HE staining). In addition, there was engorgement of the red pulp and mild haemorrhages within the lymphoid follicles and the periarteriolar lymphoid sheaths. Wild boar showed a similar lesion pattern but with a slightly lesser severity.

In the thymus (Figure 8E), immunolabelled macrophages (red arrows), tingible body macrophages (red arrowheads) and occasional lymphocytes (black arrowheads) were evenly distributed in the cortex, medulla and the corticomedullar junction, being especially numerous in pig DP39, and generally more abundant in pigs compared to wild boar. Occasionally, antigen was also detected in stellate cells, consistent with reticuloepithelial cells, in the corticomedullary junction and medulla (blue arrowhead). Microscopically, the lesions observed in pigs consisted of mild lymphoid depletion, moderate karyorrhexis and a high number of tingible body macrophages (Figure 8E, HE staining, red arrowheads). Thymuses of the wild boar assessed at humane endpoint also showed similar lesions, but to a lesser extent.

In the bone marrow in pigs, there was a moderate number of immunopositive cells, primarily myeloid cells (Figure 8F). A similar pattern was observed in wild boar, but with lower numbers of labelled cells. However, no specific lesions were evident in any of the animals evaluated, apart from the presence of occasional cell debris and a mild increase in apoptotic or cloud-like nuclei within megakaryocytes in pigs and, to a lesser degree, in wild boar.

Finally, a moderate number of immunolabelled macrophages was observed in the dermis of the dorsal skin samples taken from pigs, which were more prominent in the perivascular mononuclear infiltrates (Figure 8F, red arrows). Intravascular monocytes and capillary endothelial cells (Figure 8F, green arrows) stained for viral antigen were also detected. The wild boar showed only occasionally immunolabelled cells, mainly macrophages. Along with moderate perivascular mononuclear infiltrates, primarily consisting of lymphocytes and occasional macrophages, mild vasculitis was also observed in pigs. This lesion was especially noticeable in pig DP40 (Figure 8F, HE staining). In comparison, wild boar skin showed no specific lesions, and histopathological scores differed significantly (*p* = 0.033).

## Discussion

Comparative histopathological evaluations of tissue samples collected from pigs and wild boar inoculated intranasally with the highly virulent ASFV genotype II strain, “Armenia 2007”, revealed differences in the occurrence and distribution of viral antigen, as well as in the progression of lesions at tissue level between the two subspecies.

In wild boar, detection, increase and dissemination of viral antigen occurred earlier than in pigs. The organs of the oronasal tract, particularly the MRPLN and submandibular lymph node, were shown to be the main locations for virus replication as early as 3 dpi. Quantity of viral antigen increased in subsequent days, which was associated with local virus proliferation. The MRPLN was also the first location in which the viral antigen was detected in pigs, albeit at 5 dpi. Interestingly, despite the route of infection and the high dose used, viral antigen was only occasionally visualised in the nasal mucosa of a wild boar at 5 dpi. It was not observed in the tonsils of wild boar nor pigs at 3 dpi and was only sporadically observed at this site in one wild boar at 5 dpi. Viral antigen was also detected at 5 dpi in the lungs of wild boar and occasionally in the tracheobronchial LN, but not in pigs.

These results highlight the importance not only of MRPLN in early virus replication after intranasal infection prior to viraemia, as has been suggested previously [25, 28], but also of the submandibular LN. The role of the latter as a primary, albeit minor, target organ for virus replication has also been reported previously [28–31]. Our results, however, contrast with other studies in which the tonsils are considered to be the usual route of entry and replication of ASFV [29–33]. They instead support the hypothesis that after intranasal inoculation, ASFV can replicate in cephalic lymph nodes without first passing through the tonsils [28]. In addition, the nasal mucosa, lungs and tracheobronchial lymph nodes do not appear to play a significant role in the initial entry and proliferation of the virus following intranasal inoculation, which is consistent with other studies [25, 28]. These findings could explain why, in the current experimental infection [25] as well as in previous experiments [18, 23, 24], the viral genome was either undetectable or present at very low levels up to 5 dpi in oronasal swabs taken from wild boar and pigs that were infected oronasally with high and moderate virulence genotype II isolates. Our results also support studies suggesting that the potential for virus transmission through nasal secretions would not be efficient in early stages of infection [34], a fact that should be considered when using oronasal samples for diagnostic sampling.

Similarly, no viral antigen was detected in any of the intestinal organs or regional lymph nodes that were examined up to 5 dpi, despite the presence of mucosa-associated lymphoid tissue in these organs in both wild boar and pigs. These results also suggest that the intestinal tract may play a minor role as a portal of entry and replication for ASFV. This in turn would explain the undetectable or low levels of viral genome detected in faecal swabs taken from experimentally infected wild boar and pigs in the present [25] and previous studies [18, 23, 24].

Although none of the infected pigs euthanised up to 5 dpi became viraemic, moderate levels of viraemia were detected in one infected wild boar euthanised at 3 dpi, and higher levels in infected wild boar euthanised at 5 dpi [25]. High viraemia levels occurred concurrently with the appearance of viral antigen in the spleens of these wild boar, but not in the pigs. Both viraemia and the presence of the virus in the spleen are considered to be signs of the virus spreading throughout the body, which was deferred in pigs. Therefore, a generalised spread of the virus was evident in wild boar, but not in pigs, at 5 dpi when the viral antigen was detected by immunohistochemistry in multiple organ systems. This is consistent with the detection of viral genome in multiple tissues in our previous study [25]. In wild boar, viral antigen was not only detected in the oronasal tract, but also in the respiratory, hepatobiliary and urinary tracts. The spleen, lung and liver are organs with high resident macrophage populations and are highly susceptible to ASFV infection [35–37]. Virus replication mainly occurs in these macrophage populations, and secondarily in other cell types such as hepatocytes, endothelial cells, epithelial cells and reticular cells [27]. Replication in these organs would contribute to increased viraemia and highlights the role of these organs as secondary sites of virus replication following virus spread.

Our findings show that following intranasal inoculation of ASFV, initial replication occurs in the lymph nodes of the upper oronasal tract prior to viraemia, particularly in the medial retropharyngeal and submandibular lymph nodes. The efferent lymphatic vessels from both lymph nodes converge in the tracheal duct, which then discharges into the brachiocephalic vein, thus returning lymph to the bloodstream [38]. The virus therefore could use this pathway to reach secondary replication sites such as the spleen, liver and lungs, either within monocytes, attached to the surface of erythrocytes or freely within the bloodstream as proposed previously [12, 28, 39]. Replication of the virus in these secondary organs concurrently with replication in the primary sites mentioned would contribute to the onset and increase of viremia.

Histopathological lesions and their severity increased as the experiment progressed. However, microscopic changes, predominantly haemorrhagic lesions and characteristic findings of lymphoid tissue destruction such as pyknosis and cellular fragmentation of mononuclear cells [12, 13, 39], only emerged or became evident after detection of viral antigen. Thus, cell destruction was first observed in the MRPLN and to a lesser extent in the submandibular LN of wild boar at 5 dpi in parallel with an increased presence of infected macrophages. Therefore, the replication of the virus in these target cells may induce their destruction, as well as that of bystander cells such as lymphocytes. This occurs via indirect mechanisms induced by proinflammatory cytokines secreted by infected macrophages, resulting in an initial mild tissue injury in lymphoid tissue [35, 40–42]. Notably, haemorrhagic lesions in the submandibular LN of pigs euthanised at 5 dpi occurred in the absence of infected cells, but infected cells were observed in the nearby MRPLN. Such haemorrhages could have originated from the activation and disruption of endothelial cells, caused by an increase in intravascular cellular debris or increased vascular permeability induced by chemical mediators generated by other injured organs [12, 43].

Both wild boar and pigs that reached the humane endpoint at 6 and 9 dpi, respectively, exhibited a notable increase in the number of cells immunolabelled against viral antigen, as well as in the presence and severity of histopathological lesions in all tested organs. In addition, virus antigen and histopathological scores at the human endpoint were generally higher in pigs than in wild boar in the studied organs, even though the appearance and increase of immunolabelled cells and the appearance and progression of lesions occurred later in pigs. Thus, the lower severity and extent of lesions in wild boar were evidence of their lower tolerance of tissue damage prior to reaching the humane endpoint. This demonstrates their greater susceptibility to and lower resistance against the ASFV, characteristics previously noted in other studies [18, 23, 25, 26].

The mechanisms underlying these differences, which likely involve both viral and host factors, constitute a huge knowledge gap. Wild boar exhibit higher levels of genetic diversity than domestic pigs [44, 45], although differentiation between the two subspecies relies on only a few genetic markers [46]. It has been suggested that the innate immune response is key to controlling levels of ASFV replication and pathogenesis in different infected hosts. ASFV may be more effective in evading innate responses in domestic pigs and wild boars compared to wild African suids, leading to disease. Conversely, host genetic factors, as is likely the case in wild African suids, may reduce the activation of potentially harmful responses, thereby controlling the replication of the virus and reducing the appearance of clinical signs and tissue damage [47]. Other studies have pointed to differences in T-cell responses that may explain some of the differences in ASF progression in wild boar and pigs, although the authors did not elucidate any mechanism of protection or resistance against ASFV [48, 49]. The influence of genetic, immunological and virological factors on host susceptibility and resistance is a question that will need to be addressed in future studies. The higher susceptibility to and lower tolerance of tissue damage in wild boar compared to pigs should also be taken into account during vaccine trials, in order to avoid inaccurate assessments resulting from the occurrence of only moderate pathology in wild boar. This emphasises the need to improve our understanding of ASF in wild boar to develop vaccines that are specifically designed to control and eradicate the disease in wild boar. Comorbidities, including parasitic diseases, nutritional discrepancies and environmental stressors present in field conditions, should also be considered as factors that will make this task difficult.

To conclude, we demonstrated that viral antigen in tissues and histological lesions were detected earlier in wild boar than in pigs after intranasal inoculation with ASFV, with MRPLN and submandibular lymph nodes being among the earliest sites for virus replication. Virus antigen and histopathological scores at the human endpoint were lower in wild boar than in pigs, even though the appearance and increase of viral antigen in tissues, the progression of lesions and the humane endpoint occurred earlier in wild boar. Thus, the lower tolerance of tissue damage prior to reaching the humane endpoint demonstrated the higher susceptibility and lower resistance of wild boar to ASFV. The mechanisms behind these differences remain unclear. These findings should be considered if ASF candidate vaccines intended to wild boar are evaluated in pig models, as surrogates for wild boar. The results from this study also provide information on co-localisation of the virus with lesions at the cellular and tissue level and complement previously published data on macroscopic lesions and virus presence in the same animals.

## Supplementary Information

**Additional file 1** **Summary of histopathological and virus antigen scores in pigs and wild boar.**

## Data Availability

The datasets used and/or analysed during the current study are available from the corresponding author upon reasonable request.

## References

[1] Alonso C, Borca M, Dixon L, Revilla Y, Rodriguez F, Escribano JM (2018) ICTV virus taxonomy profile: Asfarviridae. J Gen Virol 99:613–614 29565243 10.1099/jgv.0.001049 PMC12662184

[2] Fauquet CM, Mayo MA, Maniloff J, Desselberger U, Ball LA (2005) Virus taxonomy: VIIIth report of the International Committee on Taxonomy of Viruses: Academic Press.

[3] Gallardo MC, AdlT R, Fernández-Pinero J, Iglesias I, Muñoz MJ, Arias ML (2015) African swine fever: a global view of the current challenge. Porc Health Manag 1:21 10.1186/s40813-015-0013-y PMC5382474 28405426

[4] Blome S, Gabriel C, Dietze K, Breithaupt A, Beer M (2012) High virulence of African swine fever virus caucasus isolate in European wild boars of all ages. Emerg Infect Dis 18:708 22469497 10.3201/eid1804.111813 PMC3309674

[5] Mason-D’Croz D, Bogard JR, Herrero M, Robinson S, Sulser TB, Wiebe K, Willenbockel D, Godfray HCJ (2020) Modelling the global economic consequences of a major African swine fever outbreak in China. Nat Food 1:221–228 33634268 10.1038/s43016-020-0057-2 PMC7116817

[6] Sanchez-Cordon PJ, Montoya M, Reis AL, Dixon LK (2018) African swine fever: a re-emerging viral disease threatening the global pig industry. Vet J 233:41–48 29486878 10.1016/j.tvjl.2017.12.025 PMC5844645

[7] Dixon LK, Stahl K, Jori F, Vial L, Pfeiffer DU (2020) African Swine Fever Epidemiology and Control. Annu Rev Anim Biosci 8:221–246 31743062 10.1146/annurev-animal-021419-083741

[8] Urbano AC, Ferreira F (2022) African swine fever control and prevention: an update on vaccine development. Emerg Microbes Infect 11:2021–2033 35912875 10.1080/22221751.2022.2108342 PMC9423837

[9] Mthombeni RF, Bastos AD, van Schalkwyk A, van Emmenes J, Heath L (2023) Phylogenomic comparison of seven African swine fever genotype II outbreak viruses (1998–2019) reveals the likely African origin of Georgia 2007/1. Pathogens 12:1129 37764936 10.3390/pathogens12091129 PMC10537866

[10] Chenais E, Depner K, Guberti V, Dietze K, Viltrop A, Ståhl K (2019) Epidemiological considerations on African swine fever in Europe 2014–2018. Porc Health Manag 5:6 10.1186/s40813-018-0109-2 PMC6325717 30637117

[11] Cadenas-Fernández E, Ito S, Aguilar-Vega C, Sánchez-Vizcaíno JM, Bosch J (2022) The role of the wild boar spreading African swine fever virus in Asia: another underestimated problem. Front Vet Sci 9:844209 35573420 10.3389/fvets.2022.844209 PMC9093143

[12] Gomez-Villamandos JC, Bautista MJ, Sanchez-Cordon PJ, Carrasco L (2013) Pathology of African swine fever: the role of monocyte-macrophage. Virus Res 173:140–149 23376310 10.1016/j.virusres.2013.01.017

[13] Blome S, Gabriel C, Beer M (2013) Pathogenesis of African swine fever in domestic pigs and European wild boar. Virus Res 173:122–130 23137735 10.1016/j.virusres.2012.10.026

[14] Guinat C, Reis AL, Netherton CL, Goatley L, Pfeiffer DU, Dixon L (2014) Dynamics of African swine fever virus shedding and excretion in domestic pigs infected by intramuscular inoculation and contact transmission. Vet Res 45:93 25256695 10.1186/s13567-014-0093-8 PMC4189175

[15] Pietschmann J, Guinat C, Beer M, Pronin V, Tuscher K, Petrov A, Keil G, Blome S (2015) Course and transmission characteristics of oral low-dose infection of domestic pigs and European wild boar with a Caucasian African swine fever virus isolate. Arch Virol 160:1657–1667 25916610 10.1007/s00705-015-2430-2

[16] Gallardo C, Soler A, Nieto R, Cano C, Pelayo V, Sánchez MA, Pridotkas G, Fernandez-Pinero J, Briones V, Arias M (2017) Experimental infection of domestic pigs with African swine fever virus Lithuania 2014 genotype II field isolate. Transbound Emerg Dis 64:300–304 25808027 10.1111/tbed.12346

[17] Gallardo C, Nurmoja I, Soler A, Delicado V, Simón A, Martin E, Pérez C, Nieto R, Arias M (2018) Evolution in Europe of African swine fever genotype II viruses from highly to moderately virulent. Vet Microbiol 219:70–79 29778207 10.1016/j.vetmic.2018.04.001

[18] Zani L, Forth JH, Forth L, Nurmoja I, Leidenberger S, Henke J, Carlson J, Breidenstein C, Viltrop A, Höper D, Sauter-Louis C, Beer M, Blome S (2018) Deletion at the 5’-end of Estonian ASFV strains associated with an attenuated phenotype. Sci Rep 8:6510 29695831 10.1038/s41598-018-24740-1 PMC5916933

[19] Sánchez-Cordón PJ, Nunez A, Neimanis A, Wikström-Lassa E, Montoya M, Crooke H, Gavier-Widén D (2019) African swine fever: disease dynamics in wild boar experimentally infected with ASFV isolates belonging to genotype I and II. Viruses 11:852 31540341 10.3390/v11090852 PMC6783972

[20] Pikalo J, Schoder ME, Sehl J, Breithaupt A, Tignon M, Cay AB, Gager AM, Fischer M, Beer M, Blome S (2020) The African swine fever virus isolate Belgium 2018/1 shows high virulence in European wild boar. Transbound Emerg Dis 67:1654–1659 32009303 10.1111/tbed.13503

[21] Pikalo J, Zani L, Huhr J, Beer M, Blome S (2029) Pathogenesis of African swine fever in domestic pigs and European wild boar–lessons learned from recent animal trials. Virus Res 271:197614 10.1016/j.virusres.2019.04.001 30953662

[22] Rodríguez-Bertos A, Cadenas-Fernández E, Rebollada-Merino A, Porras-González N, Mayoral-Alegre FJ, Barreno L, Kosowska A, Tomé-Sánchez I, Barasona JA, Sánchez-Vizcaíno JM (2020) Clinical course and gross pathological findings in wild boar infected with a highly virulent strain of African swine fever virus genotype II. Pathogens 9:688 32842614 10.3390/pathogens9090688 PMC7559345

[23] Gabriel C, Blome S, Malogolovkin A, Parilov S, Kolbasov D, Teifke JP, Beer M (2011) Characterization of African swine fever virus Caucasus isolate in European wild boars. Emerg Infect Dis 17:2342–2345 22172247 10.3201/eid1712.110430 PMC3311204

[24] Nurmoja I, Petrov A, Breidenstein C, Zani L, Forth JH, Beer M, Kristian M, Viltrop A, Blome S (2017) Biological characterization of African swine fever virus genotype II strains from north-eastern Estonia in European wild boar. Transbound Emerg Dis 64:2034–2041 28116841 10.1111/tbed.12614

[25] Sánchez-Cordón PJ, Lean FZX, Batten C, Steinbach F, Neimanis A, Le Potier M-F, Wikström-Lassa E, Wynne F, Strong R, McCleary S, Crooke H, Gavier-Widén D, Núñez A (2024) Comparative evaluation of disease dynamics in wild boar and domestic pigs experimentally inoculated intranasally with the European highly virulent African swine fever virus genotype II strain “Armenia 2007.” Vet Res 55:89 39010163 10.1186/s13567-024-01343-5 PMC11247888

[26] Sehl J, Pikalo J, Schäfer A, Franzke K, Pannhorst K, Elnagar A, Blohm U, Blome S, Breithaupt A (2020) Comparative pathology of domestic pigs and wild boar infected with the moderately virulent African swine fever virus strain “Estonia 2014”. Pathogens 9:662 32824331 10.3390/pathogens9080662 PMC7459997

[27] Sánchez-Cordón PJ, Floyd T, Hicks D, Crooke HR, McCleary S, McCarthy RR, Strong R, Dixon LK, Neimanis A, Wikström-Lassa E, Gavier-Widén D, Núñez A (2021) Evaluation of lesions and viral antigen distribution in domestic pigs inoculated intranasally with African swine fever virus Ken05/Tk1 (genotype X). Pathogens 10:768 34207265 10.3390/pathogens10060768 PMC8234863

[28] Plowright W, Parker J, Staple RF (1968) The growth of a virulent strain of African swine fever virus in domestic pigs. J Hyg Lond 66:117–134 5239770 10.1017/s0022172400040997 PMC2130613

[29] Heuschele WP (1967) Studies on the pathogenesis of African swine fever I. Quantitative studies on the sequential development of virus in pig tissues. Arch Virol 21:349–356 10.1007/BF01241735 4300741

[30] Colgrove GS, Haelterman EO, Coggins L (1969) Pathogenesis of African swine fever in young pigs. Am J Vet Res 30:1343–1359 4894999

[31] Greig A (1972) Pathogenesis of African swine fever in pigs naturally exposed to the disease. J Comp Pathol 82:73–79 4553010 10.1016/0021-9975(72)90028-x

[32] Fernández de Marco M, Salguero FJ, Bautista MJ, Núñez A, Sánchez-Cordón PJ, Gómez-Villamandos JC (2007) An immunohistochemical study of the tonsils in pigs with acute African swine fever virus infection. Res Vet Sci 83:198–203 17258254 10.1016/j.rvsc.2006.11.011

[33] Howey EB, O’Donnell V, de Carvalho Ferreira HC, Borca MV, Arzt J (2013) Pathogenesis of highly virulent African swine fever virus in domestic pigs exposed via intraoropharyngeal, intranasopharyngeal, and intramuscular inoculation, and by direct contact with infected pigs. Virus Res 178:328–339 24076499 10.1016/j.virusres.2013.09.024

[34] Wilkinson PJ, Donaldson AI, Greig A, Bruce W (1977) Transmission studies with African swine fever virus: infections of pigs by airborne virus. J Comp Pathol 87:487–495 908773 10.1016/0021-9975(77)90037-8

[35] Salguero FJ, Ruiz-Villamor E, Bautista MJ, Sánchez-Cordón PJ, Carrasco L, Gómez-Villamandos JC (2002) Changes in macrophages in spleen and lymph nodes during acute African swine fever: expression of cytokines. Vet Immunol Immunopathol 90:11–22 12406651 10.1016/s0165-2427(02)00225-8

[36] Carrasco L, Núñez A, Salguero FJ, San Segundo FD, Sánchez-Cordón PJ, Gómez-Villamandos JC, Sierra MA (2002) African swine fever: expression of interleukin-1 alpha and tumour necrosis factor-alpha by pulmonary intravascular macrophages. J Comp Pathol 126:194–201 11945008 10.1053/jcpa.2001.0543

[37] Sánchez-Cordón PJ, Romero-Trevejo JL, Pedrera M, Sánchez-Vizcaíno JM, Bautista MJ, Gómez-Villamandos JC (2008) Role of hepatic macrophages during the viral haemorrhagic fever induced by African swine fever virus. Histol Histopathol 23:683–691 18366006 10.14670/HH-23.683

[38] Saar LI, Getty R (1982) Lymphatic system of pigs. In: Sisson S, Grossman JD (eds) Anatomy of the domestic animals, Volume 2, 5^th^ edition. Masson S.A, Barcelona, pp. 1481–1498

[39] Sánchez-Cordón PJ, Vidaña B, Neimanis A, Núñez A, Wikström A, Gavier-Widén D (2021) Pathology of African swine fever. In: Lacolina L, Penrith ML, Bellini S, Chenais E, Jori F, Montoya M, Ståhl K, Gavier-Widén D (eds) Understanding and combatting African swine fever. A European perspective. Wageningen Academic Publishers, The Netherlands, pp 87–139

[40] Salguero FJ, Sánchez-Cordón PJ, Núñez A, Fernández de Marco M, Gómez-Villamandos JC (2005) Proinflammatory cytokines induce lymphocyte apoptosis in acute African swine fever infection. J Comp Pathol 132:289–302 15893987 10.1016/j.jcpa.2004.11.004

[41] Gómez del Moral M, Ortuño E, Fernández-Zapatero P, Alonso F, Alonso C, Ezquerra A, Domínguez J (1999) African swine fever virus infection induces tumor necrosis factor alpha production: implications in pathogenesis. J Virol 73:2173–2180 9971800 10.1128/jvi.73.3.2173-2180.1999 PMC104462

[42] Ruedas-Torres I, Thi To Nga B, Salguero FJ (2024) Pathogenicity and virulence of African swine fever virus. Virulence 15:2375550 38973077 10.1080/21505594.2024.2375550 PMC11232652

[43] Villeda CJ, Williams SM, Wilkinson PJ, Viñuela E (1993) Consumption coagulopathy associated with shock in acute African swine fever. Arch Virol 133:467–475 8257301 10.1007/BF01313784

[44] Célio Alves P, Pinheiro I, Godinho R, Vicente J, Gortázar C, Scandura M (2010) Genetic diversity of wild boar populations and domestic pig breeds (*Sus scrofa*) in South-western Europe. Biol J Linn Soc 101:797–822

[45] Scandura M, Iacolina L, Apollonio M (2011) Genetic diversity in the European wild boar *Sus scrofa*: phylogeography, population structure and wild× domestic hybridization. Mammal Rev 41:125–137

[46] Lorenzini R, Fanelli R, Tancredi F, Siclari A, Garofalo L (2020) Matching STR and SNP genotyping to discriminate between wild boar, domestic pigs and their recent hybrids for forensic purposes. Sci Rep 10:3188 32081854 10.1038/s41598-020-59644-6 PMC7035276

[47] Netherton CL, Connell S, Benfield CT, Dixon LK (2019) The genetics of life and death: virus-host interactions underpinning resistance to African swine fever, a viral hemorrhagic disease. Front Genet 10:402 31130984 10.3389/fgene.2019.00402 PMC6509158

[48] Hühr J, Schäfer A, Schwaiger T, Zani L, Sehl J, Mettenleiter TC, Blome S, Blohm U (2020) Impaired T-cell responses in domestic pigs and wild boar upon infection with a highly virulent African swine fever virus strain. Transbound Emerg Dis 67:3016–3032 32530090 10.1111/tbed.13678

[49] Schäfer A, Zani L, Pikalo J, Hühr J, Sehl J, Mettenleiter TC, Breithaupt A, Blome S, Blohm U (2021) T-cell responses in domestic pigs and wild boar upon infection with the moderately virulent African swine fever virus strain ‘Estonia2014’. Transbound Emerg Dis 68:2733–2749 33630409 10.1111/tbed.14048

