# Supplementary material for: Comparative evaluation of histopathological lesions and viral antigen distribution in domestic pigs and wild boar inoculated intranasally with the highly virulent ASFV genotype II strain “Armenia 2007”
Source: Vet Res. 2026 Jan 6;57:19. doi: 10.1186/s13567-025-01701-x (PMC12849611; doi:10.1186/s13567-025-01701-x)
Supplement: Supplementary file 1 — Additional file 1 Summary of histopathological and virus antigen scores in pigs and wild boar. [file 13567_2025_1701_MOESM1_ESM.docx]

**Supplementary table 1. Summary of histopathological and virus antigen scores in pigs and wild boar**

| Domestic pigs (DP) | | | |  | Wild Boar (WB) | | | |
| --- | --- | --- | --- | --- | --- | --- | --- | --- |
| ID | **IHC scores** | **HP**  **scores** | **Day of euthanasia** |  | **ID** | **IHC scores** | **HP**  **scores** | **Day of euthanasia** |
| DP25 | 0 | 26 | 1 dpi |  | **WB44** | 0 | 17 | 1 dpi |
| DP26 | 0 | 17 | 1 dpi |  | **WB45** | 0 | 25 | 1 dpi |
| DP27 | 0 | 36 | 1 dpi |  | **WB46** | 0 | 19 | 1 dpi |
| DP28 | 0 | 21 | 2 dpi |  | **WB47** | 0 | 20 | 2 dpi |
| DP29 | 0 | 22 | 2 dpi |  | **WB48** | 0 | 24 | 2 dpi |
| DP30 | 0 | 17 | 2 dpi |  | **WB49** | 0 | 16 | 2 dpi |
| DP31 | 0 | 29 | 3 dpi |  | **WB50** | 1 | 21 | 3 dpi |
| DP32 | 0 | 23 | 3 dpi |  | **WB51** | 7 | 25 | 3 dpi |
| DP33 | 0 | 26 | 3 dpi |  | **WB52** | 1 | 20 | 3 dpi |
| DP34 | 3 | 33 | 5 dpi |  | **WB53** | 43 | 49 | 5 dpi |
| DP35 | 4 | 17 | 5 dpi |  | **WB54** | 11 | 23 | 5 dpi |
| DP36 | 1 | 25 | 5 dpi |  | **WB55** | 10 | 21 | 5 dpi |
| DP37 | 153 | 155 | 9 dpi |  | **WB56** | 99 | 85 | 6 dpi |
| DP38 | 0 | 22 | 9 dpi* |  | **WB57** | 87 | 84 | 6 dpi |
| DP39 | 153 | 180 | 9 dpi |  | **WB58** | 38 | 54 | 6 dpi |
| DP40 | 170 | 182 | 9 dpi |  | **WB59** | 91 | 74 | 6 dpi |
| DP22 | 0 | 13 | 12 dpi (control) |  | **WB41** | 0 | 18 | 12 dpi (control) |
| DP23 | 0 | 10 | 12 dpi (control) |  | **WB42** | 0 | 10 | 12 dpi (control) |
| DP24 | 0 | 21 | 12 dpi (control) |  | **WB43** | 0 | 15 | 12 dpi (control) |

Summary of histopathological and virus antigen scores in pigs and wild boar euthanised on different days after intranasal infection with ASFV genotype II strain ‘Armenia 2007’.

(ID): Animal identification; (IHC): Virus antigen scores; (HP): Histopathological lesion scores; (dpi): days post infection; *Did not reach clinical endpoint (euthanised to prevent single housing).
